# Med23 supports angiogenesis and maintains vascular integrity through negative regulation of angiopoietin2 expression

**DOI:** 10.1038/s42003-022-03332-w

**Published:** 2022-04-19

**Authors:** Yenan Yang, Qi Xiao, Jingwen Yin, Chonghui Li, Decai Yu, Yulong He, Zhongzhou Yang, Gang Wang

**Affiliations:** 1 State Key Laboratory of Genetic Engineering, School of Life Sciences and Zhongshan Hospital, Fudan University, Shanghai, 200438 China; 2 The Ministry of Education Key Laboratory of Model Animal for Disease Study, Nanjing Biomedical Research Institute, Nanjing University, Nanjing, 210061 China; 3 Molecular and Cell Biology Laboratory, Salk Institute for Biological Studies, La Jolla, CA 92037 USA; 4 State Key Laboratory of Cell Biology, Center for Excellence in Molecular Cell Science, Shanghai Institute of Biochemistry and Cell Biology, Chinese Academy of Sciences, University of Chinese Academy of Sciences, Shanghai, 200031 China; 5 Department of Hepatobiliary Surgery, the Affiliated Drum Tower Hospital, School of Medicine, Nanjing University, Nanjing, China; 6 Laboratory of Vascular and Cancer Biology, Cyrus Tang Hematology Center, Jiangsu Institute of Hematology, the First Affiliated Hospital, Soochow University, Suzhou, China; 7 Department of Cardiology, State Key Laboratory of Pharmaceutical Biotechnology, Nanjing Drum Tower Hospital, The Affiliated Hospital of Medical School of Nanjing University, Nanjing, 210093 China

**Keywords:** Cell lineage, Embryonic induction

## Abstract

The mammalian Mediator complex consists of over 30 subunits and functions as a transcriptional hub integrating signaling for tissue-specific gene expression. Although the role of the Mediator complex in transcription has been extensively investigated, the functions of distinct Mediator subunits in development are not well understood. Here, we dissected the role of the Mediator subunit Med23 in mouse cardiovascular development. Endothelial-specific *Med23* deletion caused embryonic lethality before embryonic day 13.5 (E13.5). The mutant embryos exhibited intracranial hemorrhage and diminished angiogenesis with dilated blood vessels in the head region, where the expression of Med23 was abundant at E10.5. *Med23* deficiency impaired vasculogenesis in the head region and impeded retinal angiogenesis. Knocking down *Med23* in human umbilical vein endothelial cells (HUVECs) resulted in angiogenic defects, recapitulating the vascular defects in *Med23*-mutant mice in a cell-autonomous manner. RNA sequencing in HUVECs indicated that *Med23* deficiency resulted in the interruption of angiogenesis and the upregulation of angiopoietin2 (Ang2), an inducing factor for vascular network instability. Inhibition of Ang2 partially rescued angiogenic sprouting and lumen dilation defects in tube formation assays. Collectively, our findings demonstrate that Med23 promotes angiogenesis and maintains vascular integrity, in part by suppressing Ang2 signaling.

## Abstract (web summary)

Loss-of-function analysis and RNA Sequencing shows that Mediator subunit Med23 is important for angiogenesis during mouse cardiovascular development, in part by regulating angiopoietin2, an inducing factor for vascular network instability.

## Introduction

During early embryogenesis, vascular development initiates vasculogenesis, in which hemangioblasts differentiate into nascent endothelial cells and these cells assemble to form a rough vascular plexus^1^. Furthermore, the primary capillary plexus undergoes remolding to generate an elegant, complex network via angiogenesis, a process in which new branches sprout from preexisting vascular trunks^1^. Angiogenesis involves the proliferation, migration, and maturation of endothelial cells in the primary vascular structure and nascent sprouting vessels. Mouse genetic studies have identified a variety of genes and signaling molecules that play roles in regulating angiogenesis^2^. VEGF/VEGF receptor signaling is the most robust signaling mechanism to efficiently initiate angiogenesis^1,2^. It potently drives vascular endothelial cell proliferation and migration and substantially induces angiogenesis and the formation of a complex vascular network.

Importantly, the balance of vascular sprouting and endothelial stabilization is precisely and finely coordinated in the process of angiogenesis. Under the conditions of a lack of vascular stabilizing factors, cell–cell junctions are loose between endothelial cells, resulting in fractional instability of nascent or preexisting blood vessels. Consequently, immature endothelial cells undergo withdrawal, which causes either failure of vessel formation or malformation of nascent blood vessels^2^. Angiopoietin 1 (Ang1) is an endothelial autocrine ligand that binds to Tie2, a receptor tyrosine kinase localized in the cell membranes of endothelial cells^3^. Ang1-Tie2 signaling promotes blood vessel maturation and remodels the nascent vascular network^4,5^. Mouse genetic studies have revealed that endothelial-specific disruption of *Ang1* or *Tie2* impairs angiogenesis and disrupts blood vessel stability in an endothelial-autonomous manner^6–8^. Interestingly, Ang2 has been identified as the natural antagonist of Ang1, and a genetic study has demonstrated that Ang2 plays roles opposing those of Ang1 in vascular development and the response to injury^9^. For instance, overexpression of *Ang2* in endothelial cells reduces angiogenesis and causes blood vessel dilation in early mouse embryos^9^. However, recent data in tumor biological studies have indicated that under certain circumstances, Ang2 also stimulates angiogenesis by binding to integrins^10^. Thus, Ang2 plays dual roles in antiangiogenic and proangiogenic processes depending on the distinct context. Collectively, the evidence indicates that the coordination of proangiogenic and vascular maturation signals is indispensable for angiogenesis during vascular development and postnatal vascular homeostasis, which relies on accurately regulated Ang2/Ang1-Tie2 interactions and communication.

The Mediator complex regulates diverse gene expression programs involved in multiple cellular behaviors^11^. Biochemical and cell biological studies have revealed that this complex is composed of approximately 30 evolutionarily conserved subunits and that multiple subunits favor association with specific transcription factors, providing the individual subunits with specificity in controlling distinctive gene programs and cellular functions^12^. Increasing numbers of studies have demonstrated that certain Mediator components specifically direct particular cell fates or developmental processes. For example, the Med1 subunit is well known to regulate the activities of a variety of nuclear receptors^13^, and fibroblasts isolated from *Med1*^*−/−*^ embryos exhibit insufficient differentiation into adipocytes in a PPAR-dependent manner^14^. In addition, Med1 is a suppressor of energy expenditure in skeletal muscle^15^. The Med12 subunit acts to inhibit neuronal differentiation through epigenetic silencing of neuronal gene expression^16^. The Med13 subunit plays a role in microRNA-mediated cardiac systemic energy homeostasis^17^. The Med15 subunit has been shown to be required for axis duplication and mesendoderm differentiation in *Xenopus* embryos^18^.

The Med23 subunit was initially identified as a component of the Mediator complex for Ras/Elk1 signaling to regulate cell fate decisions, cell proliferation, and migration^19–22^. Conventional *Med23* deletion in mice causes early embryonic lethality with defects in neural and cardiovascular systems, suggesting that Med23 is an important regulator in these systems^23^. Here, we generated conditional alleles for *Med23* and specifically inactivated *Med23* in either cardiac or endothelial tissues in mice. To our surprise, cardiac deletion of *Med23* mediated by both *Nkx2.5-Cre* and α-*MHC-Cre* excision had little effect on heart development. However, deletion of *Med23* in endothelial cells with *Tie2-Cre* caused early embryonic lethality with cardiovascular defects. We observed that *Med23* endothelial-specific knockout led to enlarged vessel lumen size and remodeling deficiency of the dendritic capillary plexus in the head region. Angiogenic analysis revealed that *Med23* deficiency disrupted human umbilical vein endothelial cell (HUVEC) tube formation and impaired retinal angiogenesis. By RNA-seq analysis, Ang2 was found to be upregulated upon *Med23* knockdown, and antibody neutralization or knockdown of Ang2 partially rescued the phenotype caused by *Med23* deficiency. In summary, our findings reveal a mechanism through which Med23 participates in vascular development, in part by repressing Ang2 expression.

## Results

### Endothelial Med23 is essential for early embryonic development and survival

**Fig. 1:**
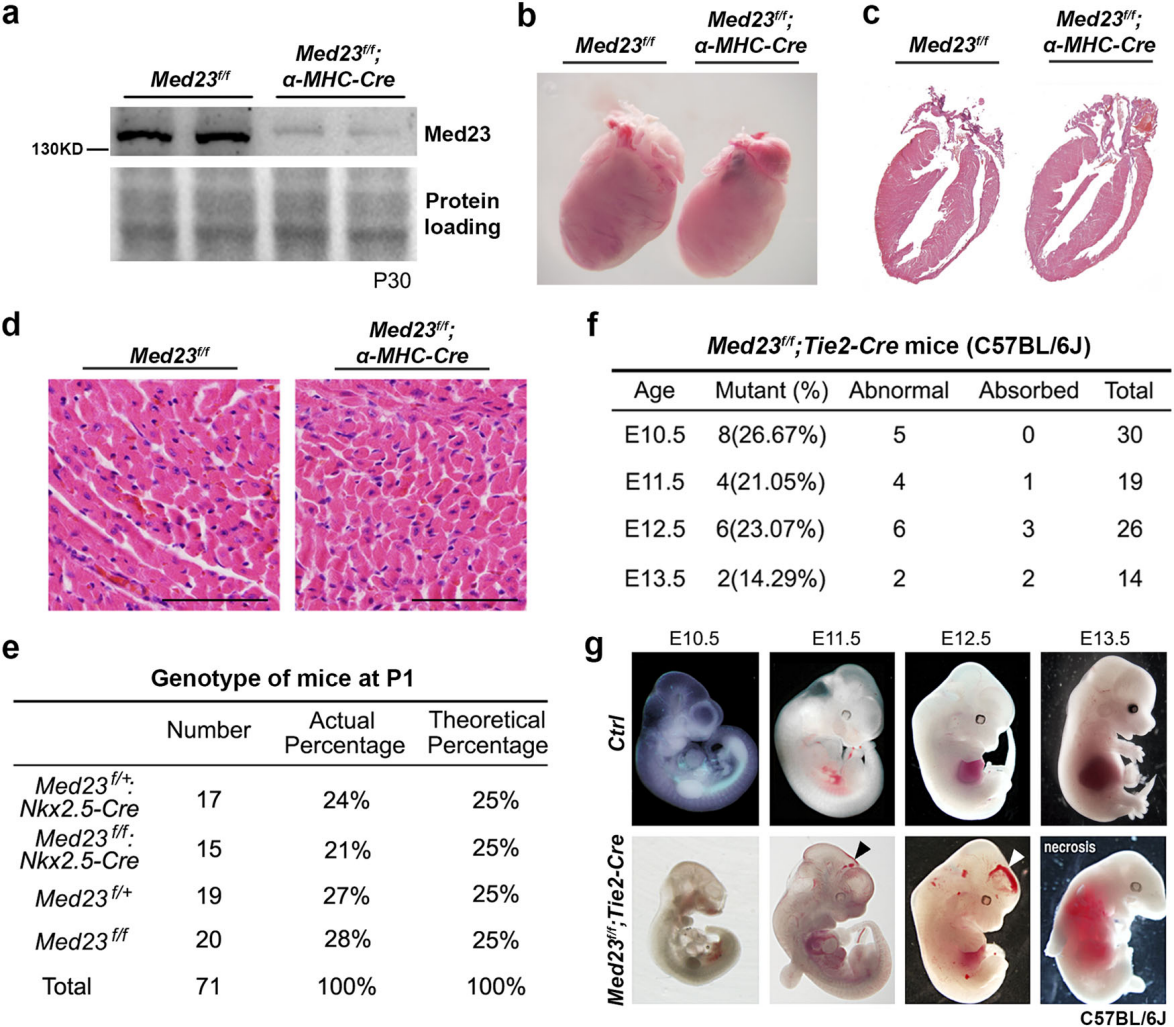
Generation and analysis of cardiac-specific and endothelial-specific *Med23*-deletion mice. **a** Western blotting analysis to confirm the deletion of Med23 in the heart mediated by α-*MHC*-Cre. Heart tissues were prepared from 1-month-old mice, and whole hearts were lysed for western blotting. **b** Morphology comparison of *Med23*-deletion hearts and control hearts. The hearts were prepared from 1-month-old mice. **c** Histological study of *Med23*-deletion and control hearts from 1-month-old mice. **d** Hematoxylin-eosin (H&E) staining of sections from *Med23*-deletion and control hearts from 1-month-old mice. Scale bar, 100 μm. **e** Gross study of the offspring of *Med23*^*f/f*^ and *Med23*^*f/+*^; *Nkx2.5-Cre* mice. The ratios of *Nkx2.5-Cre*-mediated *Med23*-deletion mice (*Med23*^*f/f*^; *Nkx2.5-Cre*) and control mice (*Med23*^*f/+*^, *Med23*^*f/f*^ or *Med23*^*f/+*^; *Nkx2.5-Cre*) were determined at P1. **f** Gross study of control and endothelial-specific *Med23-*deletion embryos under C57BL/6 J conditions. The numbers of existing and absorbed *Med23*^*f/f*^*:Tie2-Cre* embryos were determined from E10.5 to E13.5. **g** Representative views of control and endothelial-specific *Med23*-deletion mice in successive embryonic development stages. Hemorrhage was seen in the mutant mice (indicated by the arrowhead).

Conventional *Med23* deletion in mice caused early embryonic lethality with defects in the neural and cardiovascular systems before embryonic day 9.5 (E9.5), suggesting that Med23 is an important regulator in these systems^23^. To investigate the role of Med23 in cardiovascular development, we generated conditional alleles for *Med23* and specifically inactivated *Med23* in either cardiac or endothelial tissues in mice. To our surprise, cardiac deletion of *Med23* mediated by *a-MHC-Cre* had little effect on heart development, and the mice survived normally (Fig. 1a–d). Likewise, the *Nkx2.5-Cre*-mediated *Med23* cardiac-specific knockout mice in Fig. 1e generated by crossing *Med23*^*f/f*^ mice with *Med23*^*f/+*^; *Nkx2.5-Cre* mice also exhibited normal phenotype. However, the deletion of *Med23* in endothelial cells caused early embryonic lethality. Endothelial cell-specific *Med23*-knockout mice were generated by crossing *Med23*^*f/f*^ mice with *Med23*^*f/+*^; *Tie2-Cre* mice, and the resulting mice (*Med23*^*f/f*^; *Tie2-Cre*) were lost from E10.5 to E13.5 (Fig. 1f, g), but the control mice (*Med23*^*f/+*^, *Med23*^*f/f*^ or *Med23*^*f/+*^; *Tie2-Cre*) developed normally (Fig. 1g). Taken together, these findings indicate that endothelial Med23 is critically important for full embryonic development and survival.

### Med23 regulates intracranial vascular integrity and sprouting angiogenesis

**Fig. 2:**
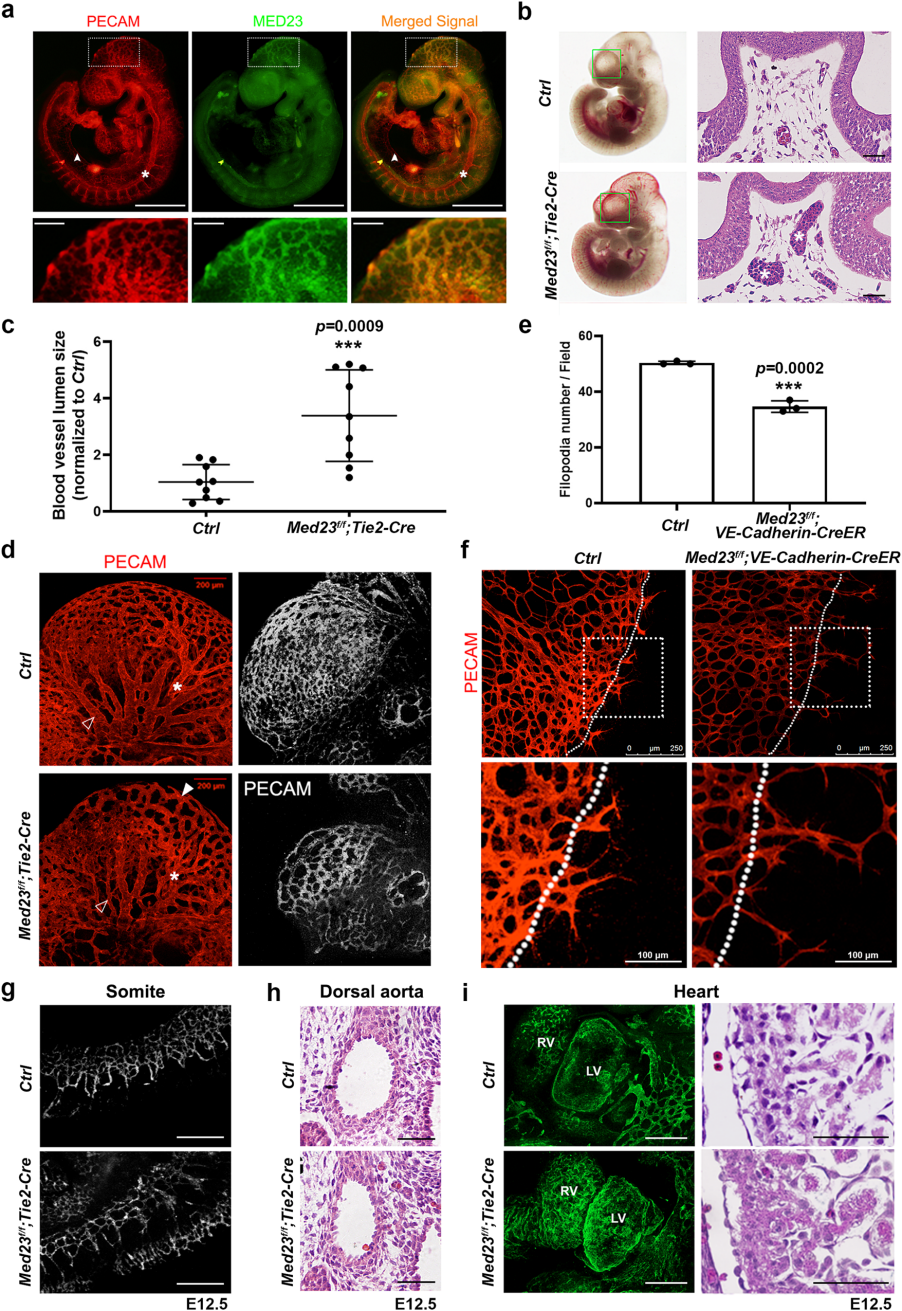
Histological analysis of vascular development in endothelial-specific *Med23*-deletion mice. **a** Whole-mount immunofluorescence staining for PECAM and Med23 in E9.5 wild-type embryos. The boxed area indicates abundant expression of Med23 in the vasculature of the head. The arrowheads and asterisks indicate the areas where the Med23 signal is weak and where Med23 does not colocalize well with PECAM. Magnified views of the boxed regions are shown under the corresponding pictures. Scale bars for the upper panel, 2.5 mm. Scale bars for the bottom panel, 0.5 mm. **b** H&E staining to analyze the lumen size of the blood vessels in the head in E9.5 embryos. The asterisks indicate blood vessels. Scale bar, 200 μm. **c** Quantitation of blood vessel lumen size in the control and *Med23*-mutant mice in **b**. Nine mouse samples from each group were used for the statistical analysis. The values are the means ± SDs. ***p < 0.001. **d** PECAM staining to display the vascular network in the heads of control and *Med23-*mutant mice (E9.5). The asterisks indicate the intracranial artery, the open arrowheads indicate the venous plexus, and the solid arrowheads mark the intracranial arterial network. A poorly developed intracranial vasculature was easily identified in *Med23-*mutant mice. Scale bar, 200 μm. **e** Quantification of filopodia in in vivo retinal angiogenesis assays. *n* = 3 for each group. The values are the means ± SDs. ***p < 0.001. **f** PECAM staining of the in vivo-generated retinal vascular network. Magnified views of the boxed regions are shown under the corresponding pictures. Scale bars for the upper panel, 250 μm. Scale bars for the bottom panel, 100 μm. **g** Immunofluorescence images of the intersomitic vasculature. Both control and *Med23*^*f/f*^*:Tie2-Cre* embryos displayed normal vascular networks. Scale bar, 200 μm. **h** Histological images of the dorsal aorta in the transaxial plane. Scale bar, 100 μm. **i** Whole-mount immunofluorescence staining of the endocardium in E12.5 embryos (left; Scale bar, 300 μm) and histological analysis of E12.5 heart sections (right; Scale bar, 50 μm). The endothelial marker PECAM was labeled in cardiac tissues by immunofluorescence staining.

To investigate the biological function of Med23 in vasculature, we focused on studying the endothelial-specific *Med23-*mutant mice. Nearly 70% of *Med23-*mutant embryos showed intracranial hemorrhage and edema starting from E10.5 (Fig. 1g). We performed immunofluorescence (IF) staining to investigate the expression pattern of Med23 in the embryo, particularly in the vasculature. IF staining revealed enriched expression of Med23 in the intracranial arterial network that was largely colocalized with the endothelial cell marker PECAM (Fig. 2a). Histological analysis revealed dilation of intracranial capillaries in the *Med23*-mutant forehead at E10.5 (Fig. 2b). Quantification of vessel lumen size indicated a significant difference between the control and *Med23* mutants (Fig. 2c). Moreover, whole-mount IF staining with an antibody against PECAM displayed vascular dilation and remodeling defects of the dendritic capillary plexus in the middle head region of the mutant (Fig. 2d).

To further investigate the effects of *Med23* loss in the endothelial system, we performed in vivo retinal angiogenesis assays, well-established assays for studying sprouting angiogenesis, in control (*Med23*^*f/f*^) and *Med23*^*f/f*^*:VE-Cadherin-Cre* mice^24^. These animals were first treated with tamoxifen (100 ng/kg/day) from Postnatal Day 1 (P1) to P3, and their retinal tissues were isolated at P5 for analysis. The vascular network in both groups displayed similar expansion capacity. However, the numbers of tip cells and filopodia at the edge of the vascular network were significantly smaller in the mutant group than in the control group (Fig. 2e). Moreover, the density of the vascular network in mutant mice was lower than that in control mice (Fig. 2f). We also observed a reduced staining signal in mutant mice compared to control mice, suggesting that the retinal vessels expressed reduced levels of PECAM protein (Fig. 2f).

Other vascular or endothelial systems in which the Med23 signal is weak or does not colocalize well with PECAM were analyzed (Fig. 2a). The intersomitic vasculature (Fig. 2g), dorsal aorta (Fig. 2h), and endocardium (Fig. 2i) were all well developed in *Med23*-mutant embryos.

Taken together, these results indicate that Med23 regulates vascular sprouting and remodeling and maintains vascular integrity in some vascular beds, especially in the head.

### *Med23* deficiency impairs angiogenesis in an in vitro model system

**Fig. 3:**
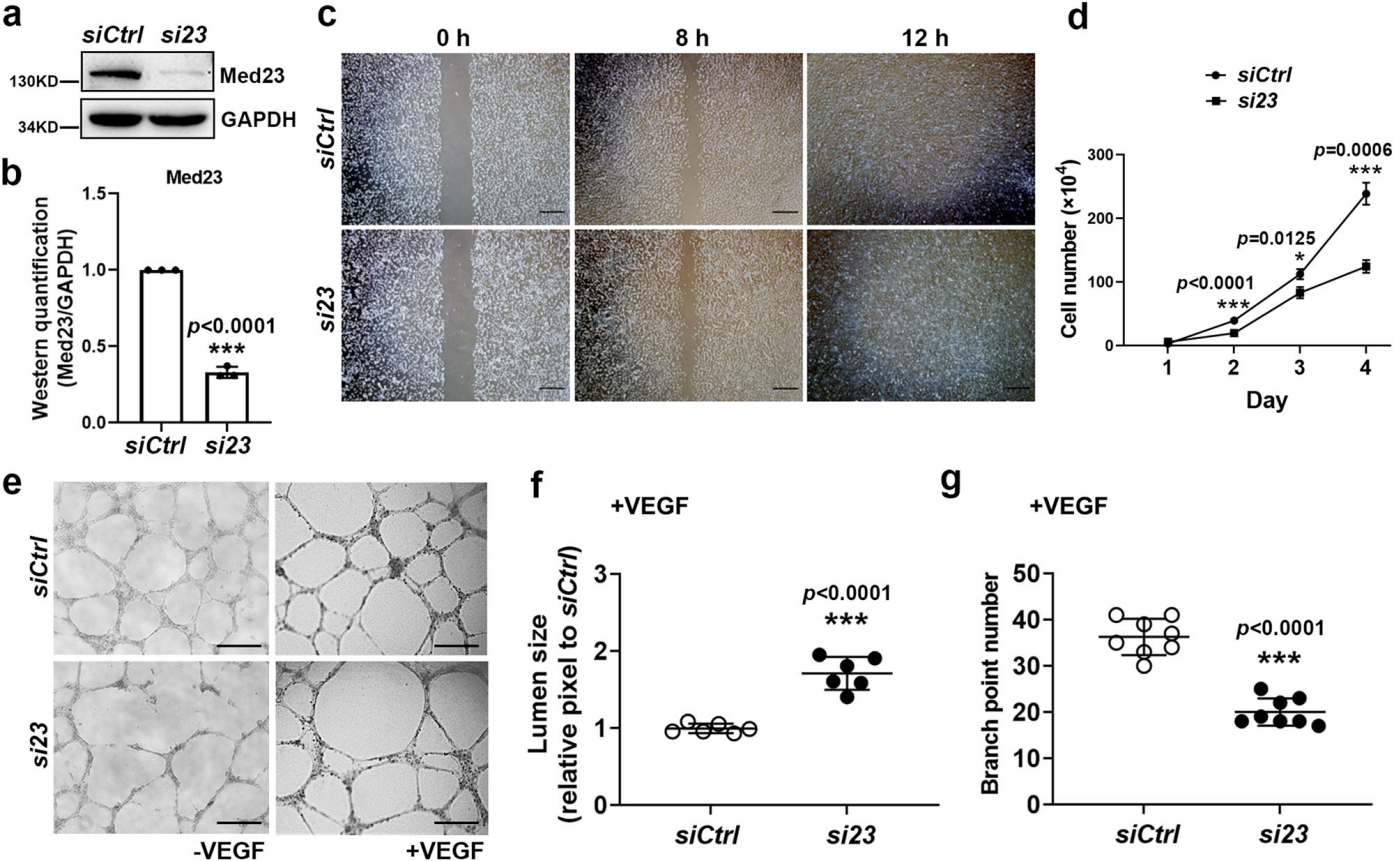
In vitro assays to evaluate the effect of Med23 on angiogenesis. **a** Western blotting to analyze the knockdown efficiency of *Med23* by mRNA silencing in HUVECs. **b** Statistical analysis of the western blotting grayscale values shown in (**a**). Three independent western blots were quantified by densitometric analysis. The value for *siCtrl* was set as 1. The values are the means ± SDs. ***p < 0.001. **c** Scratch wound cell migration assays to evaluate the impact of *Med23* knockdown on the migration of HUVECs. Images under a ×10 objective lens were captured at 0 h, 8 h, and 12 h. Scale bar, 200 μm. **d** Cell survival assays to evaluate the impact of *Med23* knockdown on the survival curves of HUVECs. *n* = 3 for each group. The values are the means ± SDs. *p < 0.05, **p < 0.01, ***p < 0.001. **e** 2D Matrigel tube formation assays with VEGF plus endothelial cell medium. Images were captured at 12 h. Scale bar, 200 μm. **f** Quantitation of the lumen sizes of the tubes formed by the control and *Med23*-knockdown HUVECs in the presence of VEGF. Each dot represents the average lumen size of one field of the corresponding group shown in (**e**). *n* = 6 for each group. The values are the means ± SDs. ***p < 0.001. **g** Quantitation of the branch points formed by the control and *Med23*-knockdown HUVECs in the presence of VEGF. Each dot represents the branch point number of one field of the corresponding group shown in (**e**). *n* = 8 for each group. The values are the means ± SDs. ***p < 0.001.

To understand the cellular mechanisms of vascular defects in *Med23*-mutant mice, we performed an in vitro angiogenesis assay using HUVECs. Retroviral-mediated *Med23* silencing (*si23*) significantly reduced Med23 protein levels in primary HUVECs (Fig. 3a, b). *Med23* deficiency had little impact on the migration of HUVECs (Fig. 3c). However, cell survival curves showed that the HUVEC survival rate decreased upon *Med23* knockdown (Fig. 3d). A 2D Matrigel tube formation assay displayed the defective angiogenic ability of *Med23*-deficient HUVECs (Fig. 3e). In the absence of VEGF, the *si23* group showed disruption in tube formation (Fig. 3e). In the presence of VEGF, both groups formed networks of tube-like structures (Fig. 3e). However, compared with control cells, *Med23*-deficient HUVECs exhibited an enlarged lumen size and reduced branch point number (Fig. 3f, g). This in vitro phenotype in the presence of VEGF mimicked the in vivo phenotype, which suggests that Med23 regulates the tube formation process of endothelial cells. Collectively, these results indicate that *Med23* deficiency impairs angiogenesis in the HUVEC model and is consistent with observations in mutant mice. These data also reveal that the vascular defects of *Med23*-mutant mice are endothelial-autonomous.

### Ang2 is upregulated by *Med23* knockdown

**Fig. 4:**
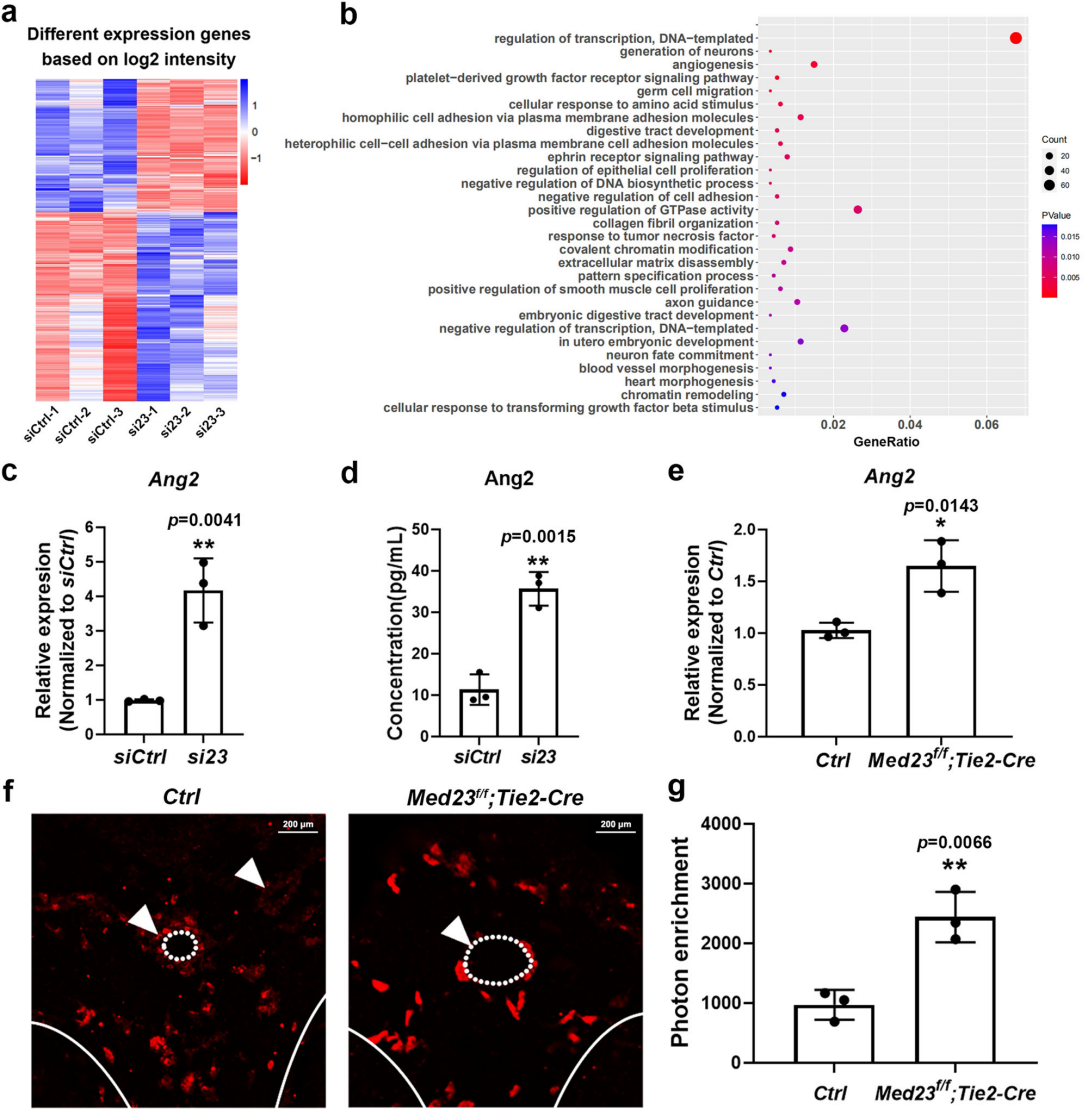
Upregulation of Ang2 by *Med23* knockdown. **a** Heatmap of RNA-seq data from HUVECs with knockdown of *Med23* or control HUVECs based on the log2 intensity (*n* = 3 for each group). **b** GO analysis of DEGs in HUVECs with *Med23* knockdown or control HUVECs, ranked by the *p* value. **c** qRT–PCR assays to validate the upregulation of *Ang2* caused by *Med23* knockdown in HUVECs based on the RNA-seq data. *n* = 3 for each group. The values are the means ± SDs. **p < 0.01. **d** Enzyme-linked immunosorbent assays (ELISAs) to validate the enhanced secretion of Ang2 upon *Med23* knockdown in HUVECs. *n* = 3 for each group. The values are the means ± SDs. **p < 0.01. **e** qRT–PCR assays to determine the upregulation of *Ang2* in the head region caused by *Med23* endothelial-specific deletion. Three embryos of the control and *Med23*^*f/f*^*; Tie2-Cre* from different litters at E12.5 were analyzed respectively. The qPCR data were normalized to the internal control *β-actin*, and the cDNAs were reverse-transcribed from the whole head RNAs. The values are the means ± SDs. *p < 0.05. **f** IF assays to determine the upregulation of *Ang2* in the head region caused by *Med23* endothelial-specific deletion at E12.5. The arrowheads indicate the positions of blood vessels. The dotted lines indicate the outlines of blood vessels. The white lines indicate the edge of samples. Scale bar, 200 μm. **g** Ang2 IF signal quantitation in (**f**). Three embryos of the control and *Med23*^*f/f*^*; Tie2-Cre* from different litters were analyzed respectively. The values are the means ± SDs. **p < 0.01.

The Mediator complex regulates gene expression to direct cell behavior. To understand how Med23 functions in endothelial cells, we performed RNA sequencing of samples prepared from control and *Med23-*knockdown HUVECs. The differentially expressed genes (DEGs) were defined as genes with an expression level fold change of more than 1.5 and a *p* < 0.05 (Supplementary Data 1). There were a total of 1139 DEGs, as shown in the heatmap (Fig. 4a). As Mediator is the key component of the transcription machinery, the regulation of transcription biological process term was the most significantly enriched term in the Gene Ontology (GO) analysis for the upregulated DEGs (Fig. 4b). In accordance with a previous report that Med23 plays an important role in neural development and acts as a regulator in cell migration and proliferation, the generation of neurons, germ cell migration, and epithelial proliferation biological process terms were enriched as well^19–23^ (Fig. 4b). As we observed, *Med23* plays an important role in angiogenesis, and the biological process of angiogenesis was significantly enriched, as expected (Fig. 4b). Among the DEGs enriched in angiogenesis, Ang2 drew our attention. As is known, Ang2 is a natural antagonist of Ang1-Tie2 signaling, and both in vitro and in vivo *Med23*-deficient endothelial cells display a rough morphological phenotype and angiogenic phenotype that is similar to the Ang1-Tie2-deficient or Ang2-overexpressing phenotype^8,9,25^, suggesting that dysregulated *Ang2* might account for the phenotype of *Med23* deletion in vascular endothelial cells.

qRT–PCR experiments confirmed the upregulation of *Ang2* in *si23*-treated HUVECs compared to the control HUVECs (Fig. 4c). In addition, Ang2 secretion was enhanced by *Med23* knockdown in HUVECs (Fig. 4d). We further determined the effect of *Med23* deficiency on Ang2 expression in vivo. qRT–PCR assays showed that the Ang2 expression level in the head region was upregulated upon *Med23* endothelial-specific depletion (Fig. 4e). Ang2 IF assays also confirmed the enhanced Ang2 signal in the head region of *Med23* endothelial-specific knockout mice in comparison with the control mice (Fig. 4f, g). In summary, Ang2 expression is upregulated by *Med23* endothelial-specific deficiency both in vitro and in vivo.

### Ang2 inhibition partially rescues angiogenic defects from *Med23* deficiency

**Fig. 5:**
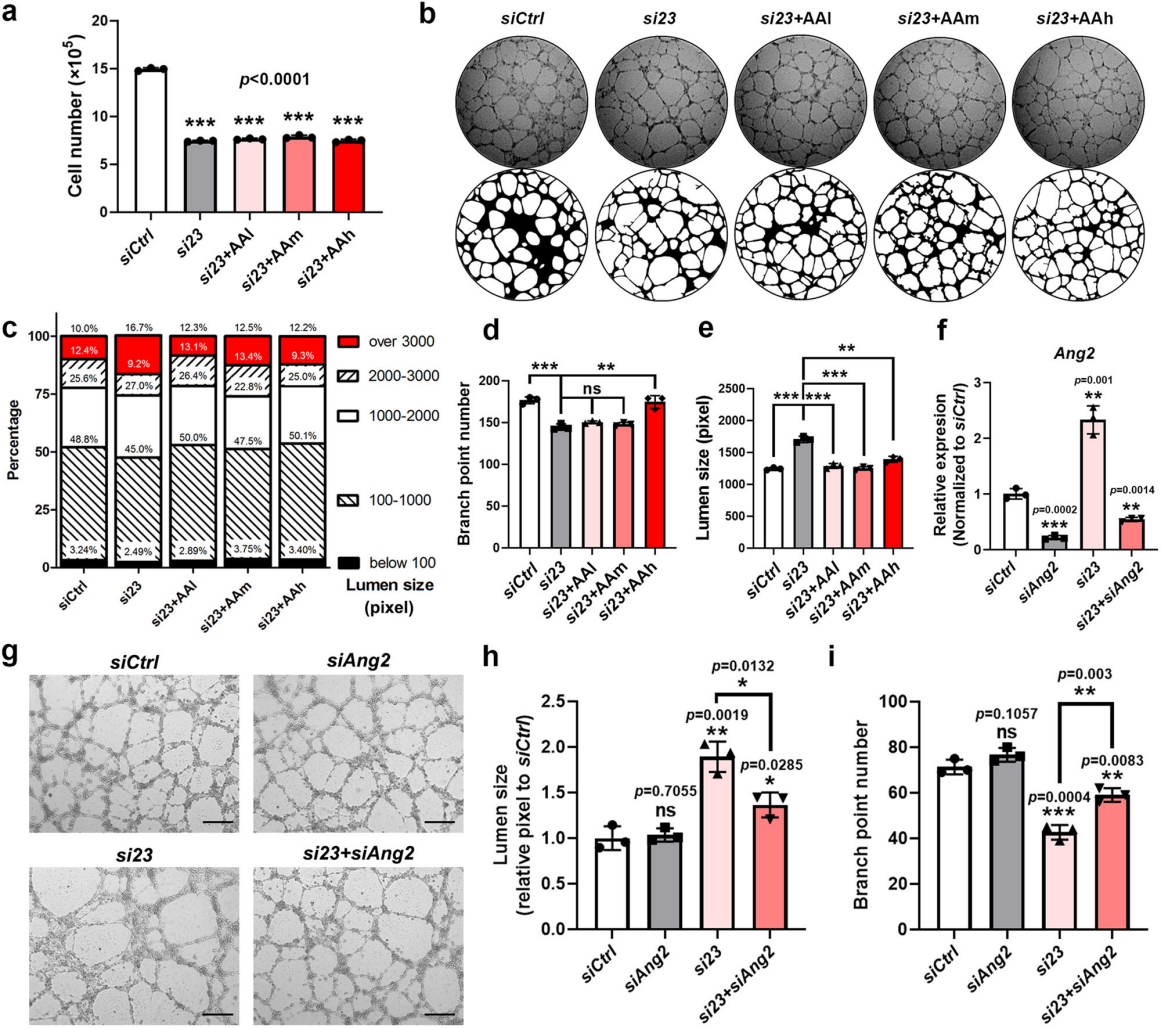
Recovery of angiogenic defects caused by *Med23* knockdown via Ang2 inhibition. **a** Cell growth assays to analyze the effects of low (0.1 ng/mL), medium (1 ng/mL), and high (10 ng/mL) doses of a neutralizing Ang2 antibody on the growth of *Med23*-knockdown HUVECs. *n* = 3 for each group. The values are the means ± SDs. ***p < 0.001. AA, Ang2 antibody. AAl, low-dose AA; AAm, medium-dose AA; AAh, high-dose AA. **b** Ang2 neutralization angiogenic assay. Images of the tube-like networks were taken under a ×20 objective lens and on the same scale. The lower panel images were well-fitted from bright-field images (the upper panel) via computer analysis. **c** Lumen size distribution analysis of the tube formation assays in (**b**). The result was calculated by fitting the image with NCBI open-source imaging software. **d** Quantitation of the branch points of the tubes in the Ang2 neutralization angiogenic assays in (**b**). n = 3 for each group. The values are the means ± SDs. **p < 0.01, ***p < 0.001. **e** Quantitation of the lumen sizes of the tubes in the Ang2 neutralization angiogenic assays in (**b**). *n* = 3 for each group. The values are the means ± SDs. **p < 0.01, ***p < 0.001. **f** qRT–PCR assays to determine the knockdown efficiency of *Ang2* in the control and *Med23*-knockdown HUVECs. *n* = 3 for each group. The values are the means ± SDs. **p < 0.01, ***p < 0.001. **g** Tube formation assays to determine the effect of *Ang2* knockdown on angiogenesis in control and *Med23*-knockdown HUVECs. Scale bar, 200 μm. **h** Lumen size quantitation of the tubes in the angiogenic assays in (**g**). *n* = 3 for each group. The values are the means ± SDs. *p < 0.05, **p < 0.01. **i** Branch point number quantitation for the tubes in the angiogenic assays in (**g**). *n* = 3 for each group. The values are the means ± SDs. **p < 0.01, ***p < 0.001.

As Ang2 is a secretory protein functioning in an autocrine or paracrine manner, to test whether enhanced Ang2 expression was causal for the angiogenic defects in *Med23*-deficient HUVECs, an anti-Ang2 antibody (AA) was administered to *si23* HUVECs for in vitro angiogenic assays. Administration of a low (0.1 ng/mL), medium (1 ng/mL) or high (10 ng/mL) concentration of AA had little effect on the proliferation or growth of *si23*-treated HUVECs (Fig. 5a). However, AA incubation indeed effectively rescued angiogenic defects in *si23*-treated HUVECs (Fig. 5b). The lumen number was increased in a dose-dependent manner upon treatment of *si23*-treated HUVECs with AA, and incubation with a high dose of AA recovered the lumen number of the *si23* group to the same level as that of the *siCtrl* group. The lumen size pattern of the AA-incubated *si23*-treated HUVEC network was modified to be similar to that of the *siCtrl* group (Fig. 5b, c). The decrease in sprouting branch points in the *si23* group was also reversed by AA treatment in a dose-dependent manner (Fig. 5d). Quantitative analysis revealed that there was no difference in sprouting branch point number between *siCtrl* cells and *si23* cells treated with a high dose of AA (Fig. 5d). Finally, AA incubation decreased the enlarged lumen size in *si23*-treated HUVECs (Fig. 5e).

For further investigation, we knocked down *Ang2* in the control and *Med23*-knockdown HUVECs. *Ang2* was confirmed to be significantly knocked down in both the control and *Med23*-knockdown HUVECs (Fig. 5f). Similar to the effect of antibody neutralization of Ang2, tube formation assays determined that knockdown of *Ang2* significantly reversed the lumen enlargement resulting from *Med23* knockdown (Fig. 5g, h). The reduction in branch point number caused by *Med23* knockdown was also partially reversed by *Ang2* knockdown (Fig. 5i).

Collectively, these results demonstrate that enhanced Ang2 levels are responsible, at least in part, for the angiogenic defects in *Med23*-deficient HUVECs, suggesting that Med23 facilitates angiogenesis by suppressing *Ang2* gene expression.

## Discussion

The Med23 subunit is considered a component of the Mediator complex that relays Ras/MAPK signaling to regulate cell fate decisions, cell proliferation, and cancer initiation^19–22,26^. Global *Med23* deletion in mice causes early embryonic lethality with defects in neural and cardiovascular systems, suggesting that Med23 is an important regulator in these systems. In this study, we demonstrated that Med23 seems to be dispensable for heart development and function but can act as an essential regulator to support angiogenesis and maintain vascular integrity. Therefore, our work has revealed the important and specific function of Med23 in vascular development.

In our RNA-seq analysis, the biological process of angiogenesis was enriched for the upregulated but not the downregulated DEGs upon *Med23* knockdown. Therefore, as a transcription cofactor, Med23 may specifically suppress the transcription of some genes. Regarding how Med23 inhibits gene transcription, a comparison of our RNA sequencing data with data in public databases revealed high similarity to PRC2 targets and H3 methylation. PRC2 and H3K9/K27 methylation suppress gene expression; therefore, Med23 may participate in these inhibitory processes to inhibit gene expression, which awaits further exploration.

Here, we found that Med23 plays a role in angiogenesis by suppressing Ang2 expression. However, Ang2 blockade in *Med23*-knockdown HUVECs only partially rescued the deficiency of *Med23*-knockdown HUVECs, indicating the existence of other effectors of Med23 in addition to Ang2 in controlling angiogenesis. Our previous finding revealed that Med23 is important in regulating alternative splicing^27^. Interestingly, a previous paper has shown that Mediator Med23 can function together with MBNL1 and MBNL2 to regulate alternative splicing events activated during endothelial cell differentiation^28^. Utilized the rMATS method^29^, we performed a differential splicing test with our RNA-seq data, and found that Med23 seems to impact 253 genes with differential alternative splicing events (Supplementary Data 2). Therefore, we postulate that Med23 may also regulate angiogenesis via alternative splicing, which awaits future investigation.

Under normal conditions, such as embryonic development and tissue growth, Ang2 is a natural antagonist of Ang1-Tie signaling, which is critical for vascular maturation and remodeling. Although the most obvious abnormality of transgenic mice overexpressing Ang2 is the apparent collapse of the endocardial lining of the heart, sectioning of *Ang2* transgenic mice has confirmed that their cranial vasculature roughly mimics the vasculature we observed in embryos lacking *Med23*, with lack of a regular dendritic capillary plexus in the head^9^. In this study, we observed profoundly enhanced *Ang2* expression in *Med23*-deficient HUVECs, and antibody neutralization against Ang2 effectively rescued the angiogenic defects. These data indicate that Ang2 plays an important role in mediating vascular defects upon Med23 deficiency. Under circumstances of tumor formation in which the Tie2 level is low, Ang2 binds to integrin and promotes angiogenesis and subsequent tumor growth and metastasis. Therefore, there is a possibility that enhancing Med23 levels may help treat tumors through the repression of *Ang2* expression. This may be a novel therapeutic strategy for tumor treatment, a possibility that should be verified in future studies.

In summary, we have identified an essential role of Med23 in angiogenesis, vascular maturation, and remodeling that is mediated, at least in part, through suppression of *Ang2* expression.

## Methods

### Animals

The experimental animal facility has been accredited by the Association for Assessment and Accreditation of Laboratory Animal Care International (AAALAC) and the Institutional Animal Care and Use Committee (IACUC) of the Model Animal Research Center of Nanjing University. All animal protocols used were approved in this study.

All mouse lines used in this study have been described previously: conditional *Med23* flox mice^30^, *a-MHC-Cre* mice^31^, *Nkx2.5-Cre* mice^32^, *Tie2-Cre* mice^33^ and *VE-Cadherin-CreER* mice^34^. The *Med23* allele was maintained on a C57BL/6 J background, and all other lines were on mixed and unspecified-strain backgrounds.

### Western blotting

Western blotting assays were performed as previously described^35^. Cells or ground tissues were sonicated in lysis buffer (50 mM Tris‑HCl, pH 7.5, containing 150 mM NaCl, 0.1 mM EDTA, 1% Triton-X‑100, 1 μg/ml aprotinin, 10 μg/ml leupeptin, and 1 mM phenylmethylsulfonyl fluoride) and centrifuged at 20,000 × *g* for 10 min. The supernatants were subjected to SDS‑polyacrylamide gel electrophoresis (SDS–PAGE) followed by Western blotting analysis with mouse anti-Med23 (BD Pharmingen, #550429, 1:1000 dilution) or anti-GAPDH (Proteintech, #HRP-60004, 1:1000 dilution) antibodies. ECL Plus Western Blotting Detection Kits (Bio-Rad, USA) and a luminescent image analyzer (Tanon, Shanghai, China) were used to visualize protein bands according to the manufacturer’s instructions.

### Ponceau S staining

After SDS–PAGE, the gels were subjected to blot transfer to a nitrocellulose membrane. The blots were then immersed in the Ponceau S solution (0.1 % (w/v) in 5% acetic acid, Sigma) for 5 min, followed by a brief rinse in distilled water to visualize the protein bands. Images were acquired using the Tanon gel imaging system. Then, a thorough rinse procedure was proceeded to completely remove the Ponceau S dye for the following immunoblotting as described above.

### Histological analysis

Hematoxylin-eosin staining was performed on formalin-fixed paraffin-embedded tissues from male or female mice of different ages. Serial 5 µm sections were stained with hematoxylin and eosin. Images were captured and processed with an Olympus FV1000 or LEICA TCS SP5II.

### Whole-mount and tissue IF staining

For whole-mount IF staining, male or female embryos at E9.5 were fixed with 4% paraformaldehyde on ice for 1–2 h and then washed for 30 min in PBS containing 0.1% Triton-X-100 (PBST) three times. Afterward, the embryos were incubated for 2 h in a blocking solution (PBS 1% Triton + 10% fetal calf serum, 0.2% sodium azide) at room temperature. Subsequently, the embryos were incubated with primary antibodies for 1–4 days at 4 °C. After washing in PBS with 1% Triton 3 times, the embryos were transferred to a secondary antibody solution and incubated for 3–4 h at room temperature. Images were captured and processed with an Olympus FV1000 or LEICA TCS SP5II.

For tissue IF staining, freshly dissected tissues from male or female mice of different ages were fixed in 4% paraformaldehyde for 48 hr and subsequently dehydrated in 30% sucrose. Then, the specimens were embedded in OCT compound (SAKURA, CA, USA) and sectioned at 10-μm thickness. The samples were blocked in PBS with 10% HS for 1 hr, incubated with primary antibodies, and then incubated with the corresponding conjugated secondary antibodies. The sections were counterstained with 4’,6-diamidino-2-phenylindole to visualize the nuclei.

The primary antibodies used in the IF assays were as follows: mouse anti-Med23 (BD Pharmingen, #550429, 1:500 dilution), rat anti-PECAM (BD Pharmingen, #550274, 1:500 dilution), and rabbit anti-Ang2 (Sigma–Aldrich, #SAB1105001, 1:500 dilution).

### Retinal angiogenesis assay

Retinal angiogenesis assays were performed as previously described^24^. Male or female *Med23*^*f/f*^*:VE-Cadherin-CreER* mice and *control* (*Med23*^*f/f*^) littermates were intraperitoneally injected with tamoxifen at a dosage of 100 ng/kg/day from P1 to P3 for postnatal vascular endothelial-specific deletion of *Med23*. Retinal tissue was harvested at P5 for IF imaging and angiogenesis analysis. Whole-mount retinas from P5 neonates were labeled with rat anti-PECAM (BD Pharmingen #550274, 1:500 dilution) in PBST. The following day, the samples were washed in PBST three times. The retinal tissue was then incubated overnight in a goat anti-rat IgG Cy3 (Jackson ImmunoResearch #106590) secondary antibody solution prepared at 1:500 in PBST. The next day, the samples were washed in PBST and mounted on glass slides. Coverslips were applied using 50% mounting glycerin.

### Culture of HUVECs

The institutional review board at Nanjing Drum Tower Hospital, Nanjing, approved this study. Samples (umbilical cords of uncomplicated human pregnancies, 38–40 gestational weeks) were obtained and used immediately after consent forms were signed.

Primary HUVECs were prepared from the human umbilical cord vein following a protocol described previously^36^. The cells were cultured in an endothelial cell medium containing 5% FBS and 1% growth supplements. Cells between the third and eighth passages were used for this study. The HUVECs were maintained in a humidified incubator at 37 °C with 5% CO_2_.

### Retrovirus infection

Retroviruses were used to establish stable *Med23-* and/or *Ang2-*knockdown HUVECs based on the manufacturer’s recommendation (Clontech) and as described previously^27^. Retroviruses were generated following the cotransfection of a recombinant pSiren-RetroQ plasmid with a pCL10A1 helper plasmid into 293 T cells using Lipofectamine 2000 (Invitrogen). The 293 T cell culture supernatants containing retroviruses were harvested 48 hr later, supplemented with 20 μg/ml polybrene, passed through a 0.45 μm filter, and then added to the HUVECs for a 1.5 hr centrifugation at 2500 rpm at 30 °C. Twenty-four hours after spin infection, HUVECs were selected with 50 μg/ml puromycin (Sigma–Aldrich). After 48 h of incubation, the medium was replaced, and the cells were used for various experiments.

### Cell migration assay

HUVECs were grown to ~70–80% confluence as a monolayer. The monolayer was scratched gently and slowly with a 1 ml pipette tip. After washing, the cells were replenished with fresh medium. Cell migration was analyzed at 0 h, 8 h, and 12 h.

### Matrigel tube formation assay

HUVECs were seeded on Matrigel-coated plates at a density of 2 × 10^4^ cells per well in 96-well dishes for 12–24 h at 37 °C. Photomicrographs were taken at ×20 and ×40 magnification. Images were processed with Photoshop software. The tube lumen size and branch point number were measured using the NIH ImageJ program.

### RNA-seq

RNA-seq was performed as previously described^35^. Total RNA was isolated with TRIzol from *Med23*-knockdown HUVECs (*n* = 3) or control cells (*n* = 3). cDNA sequencing libraries were prepared with a NEBNext® Ultra™ RNA Library Prep Kit for Illumina. The raw RNA-Seq FASTQ data were checked with FastQC (v0.1.0) and trimmed using Trimmomatic^37^ to remove adapters and poor-quality bases; reads with a quality score greater than 30 were retained and reads shorter than 50 bp were discarded. The trimmed reads were aligned to the mouse reference genome UCSC GRCh37/hg19 with HISAT2 with the default parameters^38^. The mapped results were sorted and indexed with samtools^39^ for downstream analysis. Gene and transcript quantification was performed using Cuffdiff with the parameters -b -u^40^. The transcript and gene abundance estimates are reported as the fragments per kilobase of exon per million fragments mapped (FPKM) values. To identify genes that were differentially expressed, the fold changes in each gene were calculated by dividing the average FPKM for the case by the average FPKM for the control. To avoid infinite values, a value of 0.01 was added to the FPKM value of each gene before log2 transformation. Heatmap and Gene Ontology analyses were performed using the relevant functions in R packages.

### qRT–PCR analysis

Total RNA was isolated with TRIzol and reverse-transcribed using an All-in-One First-Strand cDNA Synthesis Super Mix for qPCR (One-Step gDNA Remover) Kit (TransGen Biotech, Beijing, China). qRT–PCR was performed with 2× SYBR UltraSYBR Mix (Cwbio, Beijing, China) using a Light Cycler 480 system (Roche, Basel, Switzerland). The amplification procedure was as follows: 95 °C for 5 min followed by 40 cycles of 95 °C for 10 s and 60 °C for 20 s. The cycle threshold of each sample was used for the calculation. The expression levels of mRNA were quantified using Relative Quantification Software with *GAPDH* as an internal control.

### ELISA

ELISAs were performed using an ELISA Kit for Human Ang2 (DL Develop, Wuxi, China). Briefly, HUVECs with different treatments or control HUVECs were cultured overnight with a serum-free medium. The supernatants were obtained, and the Ang2 concentrations were quantified with ELISA kits according to the manufacturer’s instructions.

### Statistics and reproducibility

At least three independent replicates were performed for each assay. The average values from the parallel experiments are given as the means ± SDs. Analyses of differences among the groups were carried out by Student’s *t* test. Significance was defined as *p* < 0.05 (***p < 0.001, **p < 0.01, *p < 0.05).

### Reporting summary

Further information on research design is available in the Nature Research Reporting Summary linked to this article.

## Supplementary information

Supplementary Information

Description of Additional Supplementary Files

Supplementary Data 1

Supplementary Data 2

Supplementary Data 3

Reporting Summary

## Data Availability

The RNA-seq datasets generated during the current study are available in the NCBI GEO under accession number GSE186112. Source data underlying the graphs and charts in this study are included in Supplementary Data 3. Source pictures for the western blots are provided in Supplementary Figure 1.
