# Supplementary material for: Med23 supports angiogenesis and maintains vascular integrity through negative regulation of angiopoietin2 expression
Source: Commun Biol. 2022 Apr 19;5:374. doi: 10.1038/s42003-022-03332-w (PMC9019027; doi:10.1038/s42003-022-03332-w)

Supplementary Figure 1. Original western blot scans

a. Uncropped blots for Figure 1a.

Med23

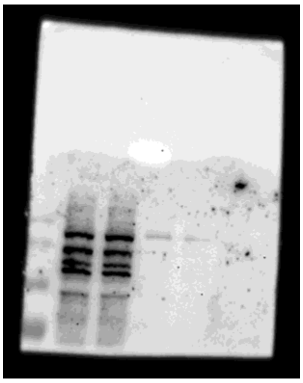

Protein Loading

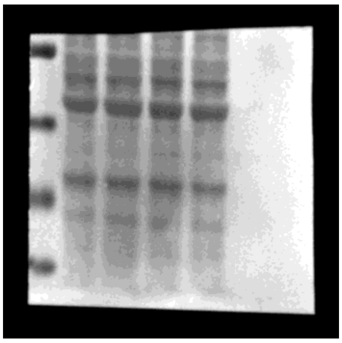

b. Uncropped blots for Figure 3a.

Med23

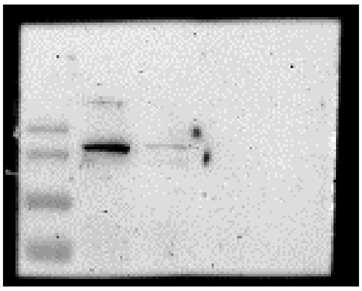

GAPDH

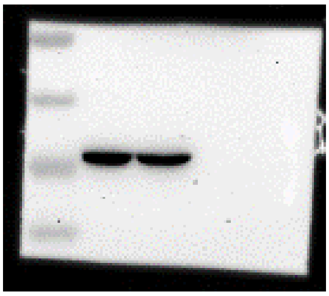

Supplement: Supplementary file 1 — Supplementary Information [file 42003_2022_3332_MOESM1_ESM.pdf]
