# Supplementary material for: Med23 supports angiogenesis and maintains vascular integrity through negative regulation of angiopoietin2 expression
Source: Commun Biol. 2022 Apr 19;5:374. doi: 10.1038/s42003-022-03332-w (PMC9019027; doi:10.1038/s42003-022-03332-w)
Supplement: Supplementary file 2 — Description of Additional Supplementary Files [file 42003_2022_3332_MOESM2_ESM.pdf]

Description of Additional Supplementary Files

File name: Supplementary Data 1
Description: : DEGs of the RNA-seq data

File name: Supplementary Data 2
Description: Alternative splicing events in the RNA-seq analysis

File name: Supplementary Data 3
Description: Source data of graphs and charts
