# Supplementary material for: Med23 supports angiogenesis and maintains vascular integrity through negative regulation of angiopoietin2 expression
Source: Commun Biol. 2022 Apr 19;5:374. doi: 10.1038/s42003-022-03332-w (PMC9019027; doi:10.1038/s42003-022-03332-w)
Supplement: Supplementary file 6 — Reporting Summary [file 42003_2022_3332_MOESM6_ESM.pdf]

nature portfolio

Corresponding author(s): DBPR COMMSBIO-21-2545B

Last updated by author(s): Mar 6, 2022

Reporting Summary

Nature Portfolio wishes to improve the reproducibility of the work that we publish. This form provides structure for consistency and transparency in reporting. For further information on Nature Portfolio policies, see our Editorial Policies and the Editorial Policy Checklist.

Statistics

For all statistical analyses, confirm that the following items are present in the figure legend, table legend, main text, or Methods section.

n/a	Confirmed	
☐	☒	The exact sample size (*n*) for each experimental group/condition, given as a discrete number and unit of measurement
☐	☒	A statement on whether measurements were taken from distinct samples or whether the same sample was measured repeatedly
☐	☒	The statistical test(s) used AND whether they are one- or two-sided<br>*Only common tests should be described solely by name; describe more complex techniques in the Methods section.*
☐	☒	A description of all covariates tested
☐	☒	A description of any assumptions or corrections, such as tests of normality and adjustment for multiple comparisons
☐	☒	A full description of the statistical parameters including central tendency (e.g. means) or other basic estimates (e.g. regression coefficient) AND variation (e.g. standard deviation) or associated estimates of uncertainty (e.g. confidence intervals)
☐	☒	For null hypothesis testing, the test statistic (e.g. *F*, *t*, *r*) with confidence intervals, effect sizes, degrees of freedom and *P* value noted<br>*Give P values as exact values whenever suitable.*
☐	☒	For Bayesian analysis, information on the choice of priors and Markov chain Monte Carlo settings
☐	☒	For hierarchical and complex designs, identification of the appropriate level for tests and full reporting of outcomes
☐	☒	Estimates of effect sizes (e.g. Cohen's *d*, Pearson's *r*), indicating how they were calculated

Our web collection on statistics for biologists contains articles on many of the points above.

Software and code

Policy information about availability of computer code

Data collection	No software was used.
Data analysis	GraphPad Prism 6.02

For manuscripts utilizing custom algorithms or software that are central to the research but not yet described in published literature, software must be made available to editors and reviewers. We strongly encourage code deposition in a community repository (e.g. GitHub). See the Nature Portfolio guidelines for submitting code & software for further information.

Data

Policy information about availability of data

All manuscripts must include a data availability statement. This statement should provide the following information, where applicable:

- Accession codes, unique identifiers, or web links for publicly available datasets
- A description of any restrictions on data availability
- For clinical datasets or third party data, please ensure that the statement adheres to our policy

RNA-seq data that support the findings of this study have been deposited in GEO with the accession codes (GSE186112). All data generated or analysed during this study are included in this published article (and its supplementary information files).

Field-specific reporting

Please select the one below that is the best fit for your research. If you are not sure, read the appropriate sections before making your selection.

☒ Life sciences ☐ Behavioural & social sciences ☐ Ecological, evolutionary & environmental sciences

For a reference copy of the document with all sections, see nature.com/documents/nr-reporting-summary-flat.pdf

Life sciences study design

All studies must disclose on these points even when the disclosure is negative.

Sample size	No sample-size calculation was performed.
Data exclusions	No data were excluded from the experiments.
Replication	At least three independent replicates were performed for each assay. All attempts at replication were successful.
Randomization	This is not relevant to our study, because randomization was not involved in our study.
Blinding	This is not relevant to our study, because group allocation was not involved in our study.

Reporting for specific materials, systems and methods

We require information from authors about some types of materials, experimental systems and methods used in many studies. Here, indicate whether each material, system or method listed is relevant to your study. If you are not sure if a list item applies to your research, read the appropriate section before selecting a response.

Materials & experimental systems

n/a	Involved in the study	
☐	☒	Antibodies
☐	☒	Eukaryotic cell lines
☒	☐	Palaeontology and archaeology
☐	☒	Animals and other organisms
☒	☐	Human research participants
☒	☐	Clinical data
☒	☐	Dual use research of concern

Methods

n/a	Involved in the study	
☒	☐	ChIP-seq
☒	☐	Flow cytometry
☒	☐	MRI-based neuroimaging

Antibodies

Antibodies used	anti-Med23 (BD Biosciences, cat No: 550429, clone: D27-1805), anti-GAPDH (Proteintech, cat No: cat No: 60004, clone: 1E6D9), anti-PECAM (BD Biosciences,cat No: 558736, clone: 390)
Validation	anti-Med23: Western blot analysis of med23.Hela nuclear extracts were probed with anti-med23 at concentrations of 5.0 ug/ml, 1.0 ug/ml. Med23 is detected at ~140 kDa.<br>anti-GAPDH: Various lysates were subjected to SDS-PAGE followed by western blot with 60004 at dilution of 1:50000 incubated at room temperature for 1.5 hours.<br>anti-PECAM: Development References :<br>1. Baldwin HS, Shen HM, Yan HC, et al. Platelet endothelial cell adhesion molecule-1 (PECAM-1/CD31): alternatively spliced, functionally distinct isoforms expressed during mammalian cardiovascular development. Development. 1994; 120(9):2539-2953. (Immunogen: Bioassay, Immunoprecipitation). View Reference<br>2. Buck CA, Baldwin HS, DeLisser H, et al. Cell adhesion receptors and early mammalian heart development: an overview. C R Acad Sci III. 1993; 316(9):849-859. (Biology). View Reference<br>3. Buck CA, Edelan JM, Buck CE, Kennedy G, Baldwin HS. Expression patterns of adhesion receptors in the developing mouse lung: functional implications. Cell Adhes Commun. 1996; 4(2):69-87. (Biology). View Reference

Eukaryotic cell lines

Policy information about cell lines

Cell line source(s)	HUVEC
Authentication	The HUVEC cells can form tube under certain condition.

Mycoplasma contamination	All cell lines tested negative for mycoplasma contamination.
Commonly misidentified lines (See ICLAC register)	No

Animals and other organisms

Policy information about studies involving animals; ARRIVE guidelines recommended for reporting animal research

Laboratory animals	The Med23 flox mice was maintained on a C57BL/6J background, Tie2-Cre and VE-Cadherin-CreER mice were on mixed and unspecified strain backgrounds.
Wild animals	The study did not involve wild animals.
Field-collected samples	The study did not involve samples collected from the field.
Ethics oversight	All animal protocols used are approved in this study.

Note that full information on the approval of the study protocol must also be provided in the manuscript.
